# Application and effect evaluation of microsurgical resection combined with intensity-modulated radiation therapy in the treatment of intracranial solitary fibrous tumor/hemangiopericytoma

**DOI:** 10.1097/MD.0000000000041336

**Published:** 2025-02-07

**Authors:** Jingcheng Jiang, Xiaoqin Qu, Han Wang, Chao Zhang, Qingshan Deng, Xiaoping Xu, Jun Qiu, Lihua Qu, Yong Yi

**Affiliations:** a Department of Neurosurgery, The Second People’s Hospital of Yibin, Yibin, China; b Department of Radiology, The Second People’s Hospital of Yibin; Clinical Research and Translational Center, Neuroimaging Big Data Research Center, The Second People’s Hospital of Yibin, Yibin, China; c Intensive Care Medicine Department, The Second People’s Hospital of Yibin, Yibin, China.

**Keywords:** microsurgery, radiotherapy, recrudescence, solitary fibrous tumor/hemangiopericytoma

## Abstract

Intracranial solitary fibrous tumor (SFT) and hemangiopericytoma (HPC) are rare mesenchymal tumors with significant vascularization, often misdiagnosed as meningiomas. Surgical resection is the primary treatment, with postoperative radiotherapy increasingly recognized for its role in improving recurrence-free survival. However, standard radiotherapy regimens remain undefined. We retrospectively analyzed clinical data from 12 patients diagnosed with SFT/HPC who underwent surgical resection and postoperative intensity-modulated radiotherapy. Clinical information, imaging findings, treatment methods, and outcomes were reviewed. Surgical resection achieved complete or subtotal tumor removal in all cases. Postoperative radiotherapy was administered to 8 patients. During follow-up, 3 patients experienced tumor recurrence, necessitating reoperation, while 1 patient died due to complications. Those who received radiotherapy showed a trend towards reduced recurrence. Surgical resection remains the cornerstone of SFT/HPC treatment, with adjuvant radiotherapy potentially improving outcomes. However, individualized treatment strategies and long-term follow-up are crucial due to the tumor’s propensity for recurrence. Further research is needed to optimize treatment approaches and enhance patient survival and quality of life.

## 1. Introduction

Intracranial solitary fibrous tumor (SFT) and intracranial hemangiopericytoma (HPC) are rare tumors derived from mesenchymal cells and are characterized by hypercellularity and significant vascularization, mostly originating from Zimmermann pericytes surrounding capillaries. The incidence of these tumors among all intracranial tumors is approximately 0.4%.^[1,2]^ According to the new World Health Organization (WHO) classification in 2016, SFT and HPC are classified as the same disease.^[3]^ SFT/HPC is easily confused with meningioma on imaging and has a higher risk of recurrence and extracranial metastasis.^[4]^ Preoperative diagnosis is challenging, and histopathological examination after surgery is the key to confirm the diagnosis.^[5]^ Prognostic determinants encompass tumor dimensions, anatomical location, extent of angiogenesis, and histopathological grade. Surgical resection is the preferred treatment method for SFT/HPC, and recent studies have shown that postoperative radiotherapy can significantly improve recurrence-free survival rate.^[6]^ However, there is no uniform standard for radiotherapy regimens targeting SFT/HPC. The heterogeneity observed in radiotherapy protocols for solitary fibrous tumors/hemangiopericytomas (SFT/HPC) is largely due to the infrequent occurrence of these neoplasms, leading to a paucity of large-scale clinical trials. This variability presents a considerable challenge for clinicians, complicating the determination of the most suitable treatment strategy for individual patients. Previous research has been deficient in data from Western China; therefore, this study seeks to augment the existing literature by assessing the efficacy of postoperative intensity-modulated radiation therapy (IMRT). This investigation may contribute to reducing the recurrence rates of SFT/HPC following surgical intervention.

## 2. Materials and methods

Clinical data.

### 2.1. General information

**Figure 1.**
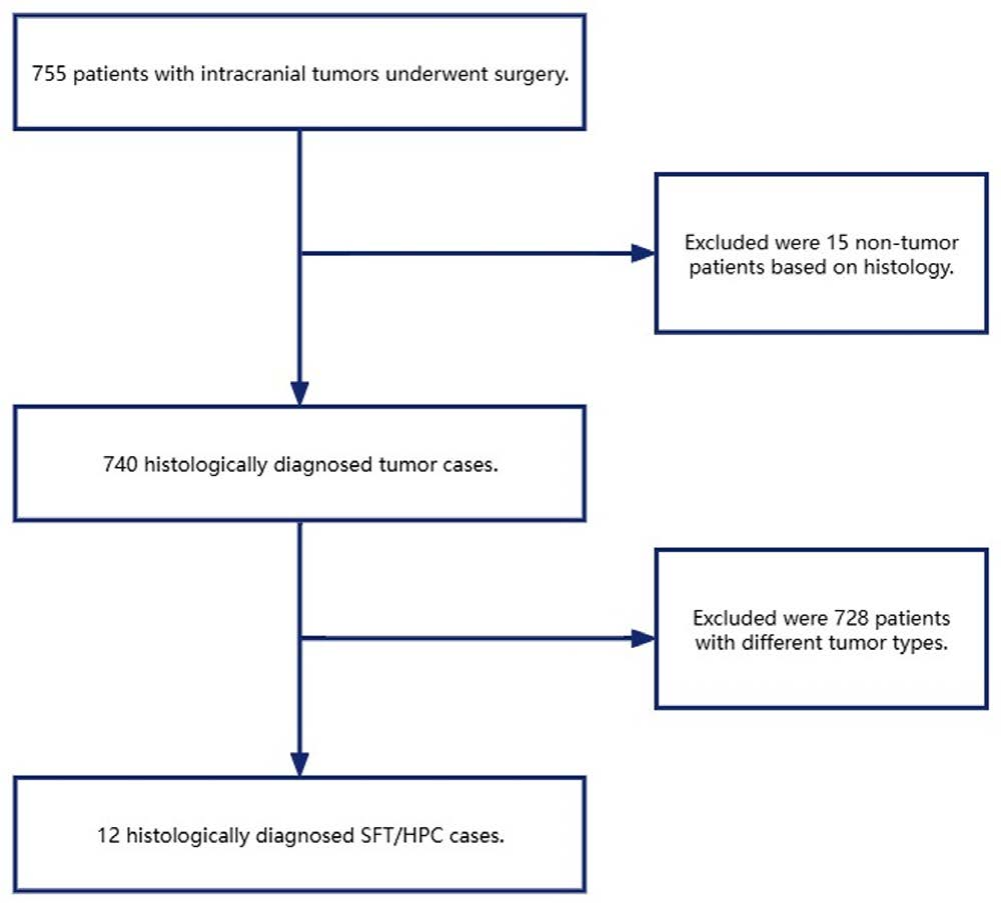
Flowchart of the study design. HPC = hemangiopericytoma, SFT = solitary fibrous tumor.

This study entails a retrospective analysis of clinical data from 12 patients diagnosed with solitary fibrous tumor/hemangiopericytoma (SFT/HPC) based on postoperative pathological examinations. The cases were collected between May 2010 and February 2022 from the Department of Neurosurgery at The Second People’s Hospital of Yibin, West China Hospital, Sichuan University (Fig. 1).

The study cohort comprised 12 patients, including 9 males and 3 females, with a mean age of 51.6 years (±16.3 years). The duration of the disease among these patients varied from 1 month to 1 year. Initial symptoms included intracranial hypertension and cranial nerve impairment in 8 patients, reduced visual acuity in 2 patients, and tinnitus in 2 patients. Preoperative scores were distributed as follows: 100 points in 3 patients, 90 points in 5 patients, 80 points in 2 patients, 60 points in 1 patient, and 40 points in 1 patient. None of the patients had a history of craniocerebral surgery or malignancy, and preoperative examinations confirmed the absence of coagulation dysfunction (refer to Tables 1, 2).

**Table 1:** Patient details.

Variables	Total (n = 12)	1 (n = 8)	2 (n = 4)	*P*	Statistic
Age, mean ± SD	51.6 ± 16.3	48.6 ± 18.2	57.5 ± 11.7	.401	0.771
Sex, n (%)				.491	Fisher
Female	3 (25.0)	3 (37.5)	0 (0)		
Male	9 (75.0)	5 (62.5)	4 (100)		
Total resect, n (%)				.333	Fisher
Yes	11 (91.7)	8 (100)	3 (75)		
No	1 (8.3)	0 (0)	1 (25)		
Grade, n (%)				.333	Fisher
Grade III	1 (8.3)	0 (0)	1 (25)		
Grade II	11 (91.7)	8 (100)	3 (75)		
Recurrence, n (%)				.222	Fisher
Yes	5 (41.7)	2 (25)	3 (75)		
No	7 (58.3)	6 (75)	1 (25)		

**Table 2:** Patients’ characteristics.

Case	Age/sex	Symptoms	Location	Tumor size	Treatment mode	Pathologic grade	Follow-up recurrence time
1	54/M	Headache for 6 mo	Right middle cranial fossa	5.9 cm × 5.8 cm × 4.5 cm	Subtotal resection	Grade III	Dead after 9 mo postoperatively
2	44/M	Right ear tinnitus for 5 mo	Right cerebellopontine angle	2.7 cm × 3.3 cm × 3.1 cm	Complete resection	Grade II	In situ recurrence at 13 mo
3	57/M	Vision decreased for 1 mo	Sellar region	1.6 cm × 1.7 cm × 1.8 cm	Complete resection + radiotherapy	Grade II	30 mo, no recurrence
4	72/M	Dizziness for 1 yr	Right temporal	3.8 cm × 3.1 cm × 3.5 cm	Complete resection	Grade II	In situ recurrence at 20 mo
5	60/M	Tinnitus 1 yr	Right middle cranial fossa	2.5 cm × 3.7 cm × 2.8 cm	Complete resection	Grade II	In situ recurrence at 21 mo
6	15/F	Headache for 2 mo	Right Temporoparietal	7.1 cm × 6.0 cm × 6.2 cm	Feeding vessel embolization + complete resection + radiotherapy	Grade II	25 mo, no recurrence
7	55/M	Right facial numbness for 3 mo	Right petroclival region	3.6 cm × 2.6 cm × 3.0 cm	Complete resection + radiotherapy	Grade II	27 mo, no recurrence
8	46/M	Headache for 1 mo	Right middle cranial fossa	4.6 cm × 3 cm × 4.5 cm	Complete resection + radiotherapy	Grade II	In situ recurrence at 25 mo
9	36/F	Left lower limb weakness for 4 mo	Right top	5.1 cm × 4 cm × 4.6 cm	Feeding vessel embolization + complete resection + radiotherapy	Grade II	22 mo, no recurrence
1 0	45M	Vision decreased for 2 mo	Left orbital apex	1.5 cm × 4.5 cm × 1.9 cm	Complete resection + radiotherapy	Grade II	24 mo, no recurrence
1 1	58/F	Dizziness for 4 mo	Left frontal	4.0 cm × 4.0 cm × 3.4 cm	Complete resection + radiotherapy	Grade II	20 mo, no recurrence
1 2	77/M	Dizziness for 1 yr	Left frontal	2.6 cm × 3 cm × 3.5 cm	Complete resection + radiotherapy	Grade II	In situ recurrence at 27 mo

### 2.2. Imaging examination

All patients completed cranial CT angiography before surgery, and 11 patients completed cranial magnetic resonance imaging (MRI) scans and contrast-enhanced examinations. The tumors were located in the orbital apex in 1 case, cerebellopontine angle in 1 case, sellar region in 1 case, middle cranial fossa in 3 cases, petroclival region in 1 case, and convex surface of the cerebral hemisphere in 5 cases. The tumor size was approximately 4.9 cm^3^ to 264.12 cm^3^, with an average of 66.35 ± 74.53 cm^3^ (Table 3).

**Table 3:** Imaging findings of patients.

Case	Tumor morphology	MRI plain scan	Enhanced MRI scan	CTA	Peritumoral conditions
1	Lobulated irregularity, solid cystic tumor, predominantly solid	Not applicable	Not applicable	Invaded right cavernous sinus, drained into right sphenoparietal sinus and cavernous sinus through venous drainage	Cavernous segment of right internal carotid artery encased, right frontotemporoparietal and basal ganglia cerebral edema
2	Oval irregular, solid tumor	Iso-T1 and iso-T2 signals were predominant, in which small cystoid-like area, long T1 and long T2 signal shadows were scattered, and DWI showed insignificant diffusion restriction	Obvious enhancement on contrast-enhanced scan	Abundant abnormal vessels within the tumor, supplied by the right anterior inferior cerebellar artery	The adjacent skull was destroyed, and the right jugular foramen was destroyed. No cerebral edema
3	Round, solid tumor	Iso-T1, slightly longer T2 signal	Enhanced scan was significantly homogeneous	Slightly compressed left cavernous sinus	Compression and absorption of the sellar dorsum, posterior clinoid process, and sellar floor without cerebral edema
4	Lobulated, solid tumor	Mixed signal	Obvious heterogeneous enhancement on contrast-enhanced scan	Abundant enhanced vessels within the tumor	No bone destruction, surrounding parenchymal edema
5	Round, solid tumor	Slightly longer T1, slightly longer T2 signal	After contrast enhancement, the lesion showed homogeneous and significant enhancement, with slight thickening and enhancement of adjacent meninges (meningeal tail sign)	It was supplied by the right middle meningeal artery and partially drained into the right transverse or sigmoid sinus	Compression and absorption of adjacent bone, significant cerebral edema
6	Lobulated, solid tumor	Slightly longer T1 and slightly longer short T2 signal, DWI showed slightly lower signal, FLAIR, SWI showed high-low signal	Nonhomogeneous obvious enhancement on contrast-enhanced scan	Multiple small tortuous vascular shadows encircling and extending around the tumor	Local bone resorption adjacent to medial occipital margin, brain edema
7	Irregular lobulated, solid tumor	Iso-T1 and slightly longer T2 mass shadows, DWI showed slightly lower signal intensity shadows, ADC showed slightly higher signal intensity shadows	Enhanced scan showed homogeneous significant enhancement	Feeding arteries and draining veins poorly visualized	No bone destruction, no brain edema
8	Round, solid tumor	Slightly longer T1 and slightly longer T2 signal shadows occupying lesions, mixed slightly higher signal intensity on DWI and FLAIR	On contrast-enhanced scan, the mass was heterogeneously and significantly enhanced, with slight thickening and enhancement of the adjacent meninges (meningeal tail sign)	Linear calcification in the margin and lesion	Adjacent bone destruction, no cerebral edema
9	Lobulated irregularity, solid tumor	Iso-T1, mixed slightly longer T2 signal	Contrast-enhanced scans showed heterogeneous significant enhancement, thickening and enhancement of the adjacent meninges, showing the meningeal tail sign	Thickened and tortuous vascular shadows were observed in the tumor, with blood supply from the branches of the right middle meningeal artery, compression of the adjacent superior sagittal sinus, and narrowing of the lumen	Slightly thinned adjacent cranial plate, no cerebral edema
10	Irregular, solid tumor	Slightly longer T1, slightly longer T2 signal	Contrast-enhanced scan showed homogeneous and obvious enhancement	Slightly compressed cavernous segment of left internal carotid artery	Enlarged optic canal left, no brain edema
11	Round, solid tumor	Iso-T1 and slightly longer T2 signal occupying lesions, small patchy long T1 and long T2 signals in the center, mixed slightly higher signal intensity in DWI and FLAIR, low signal intensity in ADC	On enhanced scan, the mass showed heterogeneous enhancement, in which small patchy non-enhanced areas were observed, surrounded by increased and thickened vascular shadows; ASL perfusion imaging lesions showed heterogeneous hypoperfusion, with a little local hyperperfusion; MRS spectroscopy showed unclear NAA in the lesion parenchyma, and Cho was significantly increased	Calcification, cystic degeneration, or necrotic foci	Skull bone thinning adjacent to cranial plate, peripheral brain edema
12	Round, solid tumor	Slightly longer T1, slightly longer T2 signal, FLAIR showed a high signal	Obvious homogeneous enhancement on contrast-enhanced scan	No vascular abnormalities	No bone destruction, large patchy cerebral edema adjacent to the left frontal lobe

ADC = apparent diffusion coefficient, ASL = arterial spin labeling, Cho = choline, CTA = CT angiography, DWI = diffusion weighted imaging (DWI), FLAIR = fluid-attenuated inversion recovery, MRI = magnetic resonance imaging, MRS = magnetic resonance spectroscopy, NAA = N-acetylaspartate, SWI = susceptibility weighted imaging, T1 = longitudinal relaxation time, T2 = transverse relaxation time.

### 2.3. Treatment methods

All patients in this group underwent tumor resection using general anesthesia. One patient with a tumor located in the sellar region underwent trans-sphenoidal endoscopic resection of the tumor, and the other 11 patients underwent craniotomy microsurgery to remove the tumor. Among them, there were 2 cases with tumors located in the convexity of the cerebral hemispheres, and preoperative images revealed rich tumor blood supply, and the tumors were removed by craniotomy after embolization of the dural feeding arteries with the Onyx liquid embolic system after a digital subtraction angiography. When dealing with skull base tumors, the subarachnoid space was explored to release cerebrospinal fluid fully after opening the dura mater and expanding the space to expose the tumor. Under microscopic guidance, the tumor base was initially addressed by disconnecting the blood supply. Sharp dissection was then carried out along the arachnoid interface to avoid damaging blood vessels and nerves. The use of the Cavitron Ultrasonic Surgical Aspirator for in situ excision of the superficial tumor was applied to reduce excessive traction on the tumor. This allows for the removal of deeper tumor tissue along the tumor cavity. In addition, neurophysiological monitoring was applied intraoperatively to identify and protect cranial nerves in real-time. For skull lesions invaded by the tumor, high-speed diamond burr grinding was used for removal, followed by complete sealing using bone wax. The defect after removing the diseased skull was occluded with its own muscles and fascia to reconstruct the skull base structure. In dealing with the convexity tumor invading the dura mater, the dura mater was completely removed, and the dura mater was repaired with a watertight suture using an artificial dura patch. All resected tumor tissues underwent pathological section examination. Postoperatively, within 6 hours, all patients underwent a follow-up head CT scan. Within 72 hours postoperatively, 11 patients underwent cranial MRI plain and enhanced scans. After the removal of sutures and wound healing, radiotherapy was initiated for 8 patients postoperatively. Treatment plan: 50 to 60 Gy in the tumor bed area, conventional fractionation, intensity-modulated radiation therapy for 30 times. Four patients did not undergo radiotherapy.

The follow-up protocol involves clinical evaluations at 3-month intervals during the first year, biannually during the second and third years, and annually thereafter. MRI scans are performed at each follow-up appointment to assess for tumor recurrence. Should imaging indicate tumor recurrence or if clinical symptoms are present, surgical intervention should be considered.

### 2.4. Statistical analysis

In statistical analysis, a continuous variable that follows a normal distribution is characterized by its mean and standard deviation. Categorical variables, on the other hand, are described by their percentage frequencies. For categorical variables, a chi-square test is employed, whereas analysis of variance (ANOVA) is conducted on continuous variables that are normally distributed. Additionally, Cox proportional hazards regression analysis is utilized to examine the independent associations between covariates and tumor recurrence.All analyses were performed using R Statistical Software (Version 4.2.2, http://www.R-project.org, The R Foundation) and Free Statistics analysis platform (Version 2.0, Beijing, China). *P* < .05 was considered statistically significant.

## 3. Results

A total of 755 participants were initially screened according to their selection and baseline characteristics. Of these, 15 individuals were excluded following postoperative histological examination, which revealed non-tumor lesions. Additionally, participants with other tumor types were excluded (n = 728; Fig. 1). After applying rigorous selection and exclusion criteria, 12 patients were ultimately enrolled in the study, comprising 9 males (75%) and 3 females (25%; Table 1).

All 12 patients underwent tumor resection, 1 subtotal tumor resection, and 11 complete tumor resections, and postoperative pathological reports indicated that 11 cases were classified as WHO Grade II, and 1 case as WHO Grade III. One patient who did not receive radiotherapy died after 9 months postoperatively due to lower respiratory tract infection and deep venous thrombosis, 3 patients experienced in situ tumor recurrence during an average follow-up of 18 months, with 2 of them undergoing a second tumor resection surgery. Among the 8 patients who received radiotherapy, 2 experienced tumor recurrence during a 26-month follow-up postoperatively and underwent a second tumor resection surgery. The remaining 6 patients were followed up for 24 months, and no tumor recurrence was observed. None of the patients experienced distant metastasis outside or inside the skull (Table 2).

In the univariate Cox regression analysis, we assessed the influence of various clinical and treatment-related variables on patient survival. The core findings are detailed as follows: total resection emerged as a significant determinant of patient survival, with an exceptionally high hazard ratio (HR) suggesting an association with markedly poor prognosis. Nonetheless, the unusually elevated HR values warrant further investigation into the accuracy of the data or the suitability of the model. The association between tumor grading and patient survival demonstrated statistical significance in the univariate analysis, suggesting that high-grade tumors correlate with a markedly poor prognosis. However, it is crucial to consider abnormal hazard ratio (HR) values. Conversely, the effect of radiotherapy on survival was not statistically significant in the univariate analysis, indicating no substantial difference in survival risk between patients who underwent radiotherapy and those who did not. This outcome may be attributed to the limited sample size in our study (Table 4).

**Table 4:** Univariable regression analysis.

Item	HR (95%CI)	*P* (LR test)
Age	0.97 (0.93, 1.02)	.238
Sex	0.21 (0.03, 1.25)	.085
Total resect	3585296118.02 (0, Inf)	.026
Grade	0 (0, Inf)	.026
Radiation therapy	2.83 (0.17, 47.15)	.478

## 4. Discussion

HPC was first documented by Stouts in 1942, with SFT being initially characterized by Murray in 1996. In 2016, the WHO reclassified these tumors as a single entity, SFT/HPC, and categorized them into Grade I, Grade II, and Grade III based on their level of malignancy.^[3]^ The resemblance of these tumors to meningiomas on imaging poses challenges for accurate preoperative diagnosis.^[2]^ The findings of our study indicate that individuals diagnosed with intracranial solitary fibrous tumor/hemangiopericytoma (SFT/HPC) are typically middle-aged males who initially present with symptoms of local nerve damage. In the advanced stages of the disease, symptoms of increased intracranial pressure may develop as a result of tumor growth.^[7]^

Although surgical resection is considered the preferred method for SFT/HPC treatment, complete tumor resection is technically more challenging compared to meningioma. The main reason for this is that the tumor is more likely to invade surrounding important structures, such as arteries, veins, nerves, skull, and dura mater (Table 3). In this study, an early case (Case 1) was not adequately assessed for tumor vascularity preoperatively due to insufficient understanding of SFT/HPC. Because the tumor invaded the cavernous sinus, the blood supply was rich and the texture was brittle, and massive bleeding occurred during the operation, resulting in the tumor not being completely removed. In cases of large SFT/HPC, the presence of dual blood supply resulting from prolonged growth may lead to significant hemorrhage during the surgical procedures of craniotomy and tumor base dissection. Therefore, precise preoperative blood supply assessment and arterial embolization are important to reduce surgical risk^[8]^ (Cases 6 and 9). There is a risk of mistakenly occluding normal vessels when embolizing intracranial vessels. The safe approach is to selectively embolize the blood vessels supplying the dura mater of the extracranial system. The current body of literature suggests that SFT/HPC can present above the intracranial tentorium and at the skull base. In instances where tumors are situated on the convex surface of the brain, employing a large skin incision and bone window is recommended. This strategy aids in the treatment of the tumor base, as well as the dura mater and skull impacted by tumor infiltration during surgical procedures. Advances in medical technology, such as 3D-printed personalized guides, real-time augmented reality (AR) technology, and neuro-navigation assistance, have significantly reduced positioning errors, enhancing surgical precision.^[9,10]^ Resection should be considered for tumors invading the dura mater and skull. To reduce the risk factors of tumor recurrence after surgery, extended resection to enhance the dura mater and invasion of the skull by the tumor is crucial. The skull base is characterized by a network of intricate blood vessels and cranial nerves. In SFT/HPC surgery of the skull base, meticulous bone grinding and cerebrospinal fluid release are crucial for enhancing tumor exposure. Microscopic intervention is essential as it provides clearer differentiation between the tumor and normal tissue, thereby reducing collateral damage. Intraoperative neurophysiological monitoring is an important tool that can identify functional areas and cranial nerves in real time and contribute to intraoperative neuroprotection, thereby improving the quality of life of patients after surgery.^[11]^ The extent of tumor resection is a direct factor affecting recurrence, and studies have shown that the extent of resection is positively correlated with postoperative recurrence and survival.^[7,12]^ When achieving Simpson Grade I resection is not feasible due to the tumor’s invasion of critical structures, subtotal resection, and adjuvant radiotherapy become important alternative options.

This study reviews the literature on SFT/HPC radiotherapy in different countries and regions^[13–20]^ and finds that patients receiving any form of adjuvant radiotherapy, including external beam radiotherapy and gamma knife radiosurgery, have a longer mean recurrence-free survival than patients not receiving radiotherapy. Patients treated with adjuvant radiation therapy after surgery benefit more than those who did not receive radiation therapy (Table 5). However, radiotherapy protocols vary among treatment centers. In this study, patients who underwent radiotherapy followed the radiation dosage protocol outlined in the Chinese expert consensus for glioma radiotherapy.^[21]^ In contrast to gliomas, SFT/HPC is classified as an extracranial lesion, necessitating a radiation treatment strategy that primarily targets the tumor bed to minimize damage to surrounding brain tissue. Reports suggest that postoperative IMRT is more advantageous in extending disease-free survival in patients compared to postoperative stereotactic radiosurgery. IMRT enables precise modulation of radiation intensity, thereby enhancing the accuracy of the radiation dose delivery. Moreover, IMRT facilitates the concentration and administration of higher radiation doses to targeted areas while concurrently minimizing exposure to adjacent healthy tissues.^[22]^ Following the guidelines outlined by the Radiation Therapy Oncology Group (RTOG), a total dose of 60 Gy was administered using intensity modulated radiation therapy over the course of 30 sessions. Additionally, efforts were made to safeguard critical organs such as the optic nerve, hippocampus, and brainstem during treatment.

**Table 5:** Literature on SFT/HPC radiotherapy.

Year	Patients, n	M/F	Tumor grade	Surgical resection	Timing of radiotherapy	Radiation therapy mode and dose	Follow-up time (mo)	Overall survival (mo)	Time to relapse (mo)	Number of recurrences
Swaminathan^[13]^	34	17/17	3 Grade I, 10 Grade II, 21 Grade III	Complete resection 23, subtotal resection 11	Perioperative	Perioperative radiotherapy, dose not mentioned	79	210	81	21/34
Lee^[14]^	85	43/42	39 Grade II, 44 Grade III	Complete resection 54, subtotal resection 31	After surgery	2D conventional radiotherapy (3 cases), 3D conformal radiotherapy (47 cases) and intensity-modulated radiotherapy (35 cases), median number of fractions 30 and median dose 60 Gy	76.9	74.6	39.2	51/85
Chenhui^[15]^	4	2/2	Not mentioned	Not mentioned	After surgery	Radiotherapy was performed in 3 cases, and gamma knife in 1 case. Number of doses not mentioned	74.6	Not mentioned	30	2/4
Yip^[16]^	5	2/3	4 Grade II, 1 Grade III	Not mentioned	After surgery	Radiation therapy (4 cases, 64 Gy, 30 fractions), Gamma Knife 1 case	75.9	Not mentioned	Not mentioned	Not mentioned
Gou^[17]^	42	25/17	22 Grade II, 20 Grade III	Complete resection 22, subtotal resection 15, not depicted 5	After surgery	Intensity-modulated radiotherapy was performed in 20 cases with a median dose of 60 Gy (range 40–63 Gy), and the number of fractions was not mentioned. Gamma knife radiosurgery was performed in 2 cases, and the single dose to the tumor margin was 11–15 Gy	96	100	15	22/42
Ghia^[18]^	39	25/14	Not mentioned	Complete resection 17, subtotal resection 16, unknown 6	After surgery	Radiotherapy 60 Gy, 30 fractions	Not mentioned	154	Not mentioned	13/39
Tsugawa^[19]^	7	3/4	Not mentioned	Not mentioned	After surgery	Gamma knife, mean edge dose 16.5 Gy, 7 patients 32 fractions	52	Not mentioned	35	2/7
González-Vargas^[20]^	7	2/5	1 Grade I, 5 Grade II, 1 Grade III	Not mentioned	Not mentioned	Not mentioned	98	Not mentioned	15.5	2/7
Our cases	8	5/3	8 Grade II, 3 Grade III	Complete resection 11, subtotal resection 1	After surgery	50 Gy −60 Gy in tumor bed area, conventional fractionation, intensity-modulated radiation therapy for 30 fractions	36	Not applicable	15	2/8

HPC = hemangiopericytoma, SFT = solitary fibrous tumor.

SFT/HPC exhibits similarities to meningiomas in conventional imaging, making preoperative diagnosis prone to confusion (Table 2). Based on our clinical experience, SFT/HPC exhibits invasiveness towards the skull. The imaging findings of skull bone resorption and destruction are significantly different from those of thickened skulls in meningiomas, which need to be discriminated based on extensive clinical experience. The application of advanced imaging techniques such as magnetic resonance spectroscopy (MRS), apparent diffusion coefficient, and positron emission tomography/computed tomography (PET/CT) can improve the accuracy of preoperative diagnosis and help physicians develop more appropriate treatment plans.^[23–26]^ Intraoperative pathological frozen sections are also critical for physicians to adjust the surgical plan.^[27]^ Given the high recurrence rate of SFT/HPC, long-term postoperative follow-up plays an important role in detecting early tumor recurrence despite total tumor resection and postoperative adjuvant radiotherapy. For recurrent tumors with surgical conditions, reoperation is a necessary option. For patients who lose the chance of surgery or are reluctant to undergo surgery again, new chemotherapeutic agents, targeted agents, and anti-tumor immunosuppressive agents are emerging.^[28]^ Pharmacological intervention has the potential to extend the duration of disease-free survival in patients,^[29,30]^ providing new possibilities for SFT/HPC treatment.

Overall, the treatment of SFT/HPC requires an individualized treatment plan based on the specific conditions of each patient. During the course of treatment, patients should be closely monitored for changes in their condition, and imaging evaluation should be performed regularly in order to detect tumor recurrence or progression in a timely manner and adjust treatment strategies. This research elucidates the central importance of achieving complete tumor resection in the treatment of solitary fibrous tumors/hemangiopericytomas (SFT/HPC) and advocates for the utilization of adjuvant radiotherapy to mitigate recurrence rates. Furthermore, the study underscores the critical significance of long-term postoperative surveillance in promptly detecting tumor recurrence. Considering the limitations of the current sample size, it is anticipated that future multicenter studies will incorporate a larger number of cases to further investigate the treatment of SFT/HPC.

## Acknowledgments

We thank the Team of Clinical Scientists for their helpful review and comments regarding the manuscript.

## Author contributions

**Conceptualization:** Jingcheng Jiang, Xiaoqin Qu, Han Wang, Chao Zhang, Lihua Qu.

**Data curation:** Jingcheng Jiang, Xiaoqin Qu, Qingshan Deng, Xiaoping Xu, Jun Qiu, Lihua Qu.

**Formal analysis:** Jingcheng Jiang.

**Investigation:** Jingcheng Jiang.

**Resources:** Jingcheng Jiang.

**Supervision:** Yong Yi.

**Validation:** Yong Yi.

**Writing – original draft:** Jingcheng Jiang, Xiaoqin Qu, Han Wang, Chao Zhang, Qingshan Deng, Xiaoping Xu, Jun Qiu, Lihua Qu.

**Writing – review & editing:** Yong Yi.
